# Amylase and lipase levels in the metabolic syndrome and type 2 diabetes: A longitudinal study in rhesus monkeys

**DOI:** 10.14814/phy2.16097

**Published:** 2024-07-02

**Authors:** Uddhav K. Chaudhari, Barbara C. Hansen

**Affiliations:** ^1^ Department of Internal Medicine, Obesity Diabetes and Aging Research Center, Morsani College of Medicine University of South Florida Tampa Florida USA; ^2^ ICMR‐National Institute for Research in Reproductive and Child Health (NIRRCH) Mumbai India

**Keywords:** amylase, diabetes, lipase, metabolic syndrome, pancreas

## Abstract

Latent associations between low serum amylase and reduced plasma insulin levels and increased adiposity have been described previously in a small study of asymptomatic middle‐aged humans. In the present study, we sought to determine the nature of such changes during the longitudinal progression from metabolically normal to overt type 2 diabetes mellitus (T2DM) in nonhuman primates (NHPs), a disease that appears to be the same in both pathophysiology and underlying mechanisms as that which most commonly develops in middle‐aged adult humans. Amylase and lipase levels were characterized in 157 unrelated adult rhesus monkeys (*Macaca mulatta*); 38% developed T2DM while under study. In all monkeys, multivariable linear regression analysis revealed that amylase could be negatively predicted by % body fat (*β* −0.29; *p* = 0.002), age (*β* −0.27; *p* = 0.005), and HbA1c (*β* −0.18; *p* = 0.037). Amylase levels were positively predicted by lipase levels (*β* = 0.19; *p* = −0.024) in all NHPs included in the study. Amylase was significantly lower in NHPs with metabolic syndrome (*p* < 0.001), prediabetes (PreDM) (*p* < 0.001), and T2DM (*p* < 0.001) compared to metabolically normal adult NHPs. Lipase increased in NHPs with PreDM (*p* = 0.005) and T2DM (*p* = 0.04) compared to normal NHPs. This is the first longitudinal study of any species, including humans, to show the dynamics of amylase and lipase during the metabolic progression from normal to metabolic syndrome, to PreDM and then to overt T2DM. The extraordinary similarity between humans and monkeys in T2DM, in pancreatic pathophysiology and in metabolic functions give these findings high translational value.

## 1 INTRODUCTION

Anatomically, the acinar cells of the exocrine pancreas, compose 85%–90% of the mass of the pancreas, and amylase, a glycolytic enzyme, and lipase, a lipolytic enzyme, are the primary exocrine enzymes of the pancreas. Exocrine pancreas acinar cells are in proximity to the cells of the islets of Langerhans, and the endocrine hormone, insulin, secreted by the islet β‐cells, and influence pancreatic endocrine functions. Insulin deficiency is associated with pancreatic atrophy and exocrine pancreatic insufficiency (Bellin et al., 2017; Terzin et al., 2014). Clinically, increases in serum amylase and lipase have been associated with acute pancreatitis (Ismail & Bhayana, 2017) and with Type 1 DM ketoacidosis (Quiros et al., 2008), whereas, low amylase and lipase have been associated with diffuse pancreatic destruction, secondary to chronic pancreatitis (Kwon et al., 2016). By contrast, serum lipase has not been consistently associated with obesity, metabolic syndrome or T2DM (Hameed et al., 2015). For instance, lipase content in pancreatic tissue was increased in adult, obese, Zucker rats (Bruzzone et al., 1984), but not in obese humans. Reports also show reduced serum lipase in patients with type 1 diabetes (Aughsteen et al., 2005), and increased serum lipase in T2DM patients (Malloy et al., 2012; Steinberg et al., 2014).

Amylase production (Korc et al., 1981; Mossner et al., 1990) and secretion (Patel et al., 2004) appear to be regulated by insulin; however, the effect of insulin resistance on amylase production is not known. In the neonatal pig model, serum amylase levels showed an inverse association with serum insulin levels from birth to 3 months of age (Pierzynowska et al., 2018). Young Zucker obese rats, exhibiting hyperinsulinemia, had increased production of pancreatic amylase, although in aged, obese rats hyperinsulinemia was associated with lower levels of pancreatic amylase and impairment of amylase gene expression (Trimble et al., 1986). Further, lower serum amylase was associated with decreased insulin levels and higher estimates of insulin resistance in middle‐aged adult humans (Muneyuki et al., 2012). In individual studies higher or lower lipase levels have been not consistently found to be associated with obesity, metabolic syndrome, T1DM or T2DM. However, a pooled meta‐analysis of 20 studies involving 20,916 participants concluded that low serum levels of amylase and lipase were significantly associated with T2D, T1D, excess adiposity, and metabolic syndrome (Ko et al., 2020). However, longitudinal trajectories of serum amylase and lipase levels during the progressive course of metabolic syndrome have not been investigated.

Middle‐aged NHPs, fed ad‐libitum on a healthy diet under laboratory conditions, frequently and spontaneously develop obesity, metabolic syndrome and T2DM (Hansen et al., 2013) with pathophysiology that is highly similar to humans. Thus, NHPs provide an ideal model for studying the longitudinal progression from metabolically normal to metabolic syndrome, PreDM, and T2DM, and for evaluation of novel therapeutic interventions. The present study aimed to evaluate serum amylase and lipase levels, with respect to their relationship to the metabolic trajectories in NHPs.

## 2 MATERIALS AND METHODS

### 2.1 Primate colony and care

Rhesus monkeys (*Macaca mulatta*) (*n* = 157; 118 male), ranging in age from 3 to 40 years, and weight 4 to 28 kg, were studied throughout their lives. All monkeys were born in breeding colonies in the United States with known age and medical history. The monkeys were housed in standard primate caging in accordance with the *Guide for the Care and Use of Laboratory Animals* (National Research Council, 2011) and were provided with consistent dietary conditions and environmental enrichment. The monkeys were ad‐libitum fed a grain‐based diet (Lab Diet 5038, PMI Nutrition International, Richmond, IN). The composition of primate diet consisted of 13% of calories by fat, 18.1% by protein, and 68.7% calories by carbohydrates. All animals were house individually in the cages. All protocols were reviewed and approved by the Institutional Animal Care and Use Committee of the University of South Florida. The colony of NHPs were studied retrospectively between 1998 and 2014 for this study.

### 2.2 Metabolic parameters

Blood samples were obtained following an overnight (16‐h) fast, under ketamine sedation (10 mg/kg) a minimum of twice per year. Parameters included: fasting plasma glucose (FPG) (glucose oxidase method, Beckman Autoanalyzer 11, Fullerton, California); fasting plasma insulin (FPI) (radioimmunoassay, RIA method of Millipore, St Charles, MO), blood chemistry (Antech Diagnostic, Tampa, FL; or the Abaxis Piccolo analyzer, Abaxis, Union City, CA), lipid profiles (VAP by Atherotech, Birmingham, AL; or Abaxis Piccolo analyzer), and estimated % body fat (tritiated water dilution method (Bodkin et al., 1993); or dual‐energy x‐ray absorptiometry (DEXA)).

The euglycemic hyperinsulinemic clamp procedure was performed under ketamine anesthesia (10 mg/kg) following a 16‐h fast. A priming dose of insulin, followed by continuous insulin infusion was administered (400 mU/m^2^ body surface area/min). This dose produced maximal insulin‐stimulated glucose uptake in both lean and obese monkeys with a coefficient of variation of 10 ± 0.5% (Bodkin et al., 1989). A variable infusion of glucose (20%) maintained plasma glucose at approximately 80 mg/dL, and the resulting insulin‐stimulated glucose uptake measurement (M‐rate) was corrected for fat‐free mass (FFM) (Bodkin et al., 1993).

### 2.3 Metabolic status groups

Serum chemistries of the monkeys, all of which had a minimum of two determinations per year for ≥3 years, were sorted based on their metabolic status. Numerical phasing from young healthy to overt T2DM, as defined by Hansen et al (Hansen & Bodkin, 1986). was used to characterize the sequential nature of the metabolic trajectory as follows: normal (*n* = 51; phases 1–3), metabolic syndrome (*n* = 44; phases 4–6), PreDM (*n* = 27; phase 7), and T2DM (*n* = 35; phase 8). Individual monkeys could appear in more than one of these phase groups if they progressed. Monkeys that developed metabolic syndrome were identified by three or more of the following criteria (Hansen & Bray, 2008): obesity (males >13.5 kg, females >8.5 kg), body fat >22%, TG ≥150 mg/dL, and glucose disappearance rate ≤2.5%/min (Jen & Hansen, 1988; Zhang et al., 2011). PreDM included the additional criteria of FPG ≥80 and/or HbA1c ≥5.5. T2DM was defined using the human definition set forth by the ADA (American Diabetes Association, 2019) of FPG ≥126 mg/dL on two consecutive occasions or an HbA1c >6.5 (American Diabetes Association, 2019).

### 2.4 Subgroups based on age of T2DM onset

Fifteen monkeys developed T2DM between the ages of 11 and 20 years and were followed prospectively from normal to T2DM. Seven of the 15 monkeys developed T2DM in early onset (14.6 ± 0.4 years (mean ± SE), range 13.8–16.3 years) and eight had a late onset of T2DM (18.6 ± 0.3 years, range 17.4–20 years).

### 2.5 Statistical analysis

**FIGURE 1:**
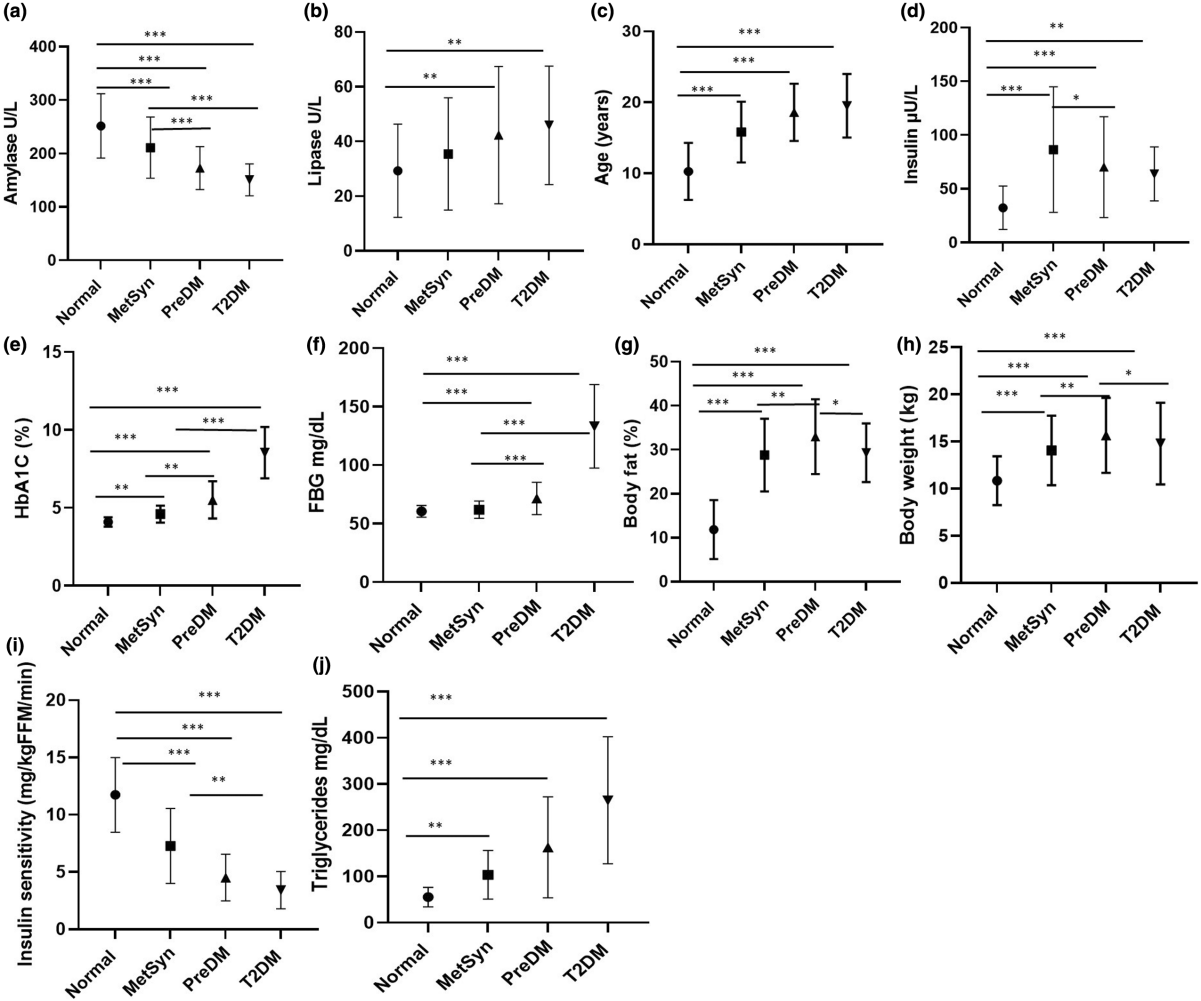
Amylase, lipase and other metabolic characteristics in normal, metabolic syndrome (MetSyn), Pre‐diabetic (PreDM) and T2DM NHPs. Amylase (a), Lipase (b), Age (c), fasting insulin (d), HbA1c (e), FBG (f), body fat % (g), body weight (h), insulin sensitivity (i) and triglycerides (j). Amylase (a) and insulin sensitivity (i) decline with progression of normal to T2DM. *p < 0.05; **p < 0.01; ***p < 0.001. Mixed model linear regression, multiple group comparison by Bonferroni post hoc test. (Normal *n* = 51; MetSyn *n* = 44; PreDM *n* = 27; T2DM *n* = 35).

Data were expressed as mean ± SD. Linear mixed‐effect model (fixed effects) were used to compare differences between amylase and lipase levels across the different ages and to compare the differences between the normal, metabolic syndrome, PreDM and T2DM groups. The Brown–Forsythe test was used to test homogeneity of variances and the Bonferroni post hoc test was used to identify the pairs of groups that contributed to the overall statistically significant difference between groups.tewise multivariable linear regression analysis was used to predict the association of amylase level with % body fat, age, lipase, HbA1c, fasting blood glucose, body weight, fasting insulin, HDL cholestrol and triglycerides. Mixed method two‐way ANOVA was used to compared early onset and late onset T2DM longitudinal data. SPSS Statistics 25 software was used for stepwise multivariable linear regression data analysis (IBM Corp, New York), however Graphpad Prism version 9.3.1 software was used for linear mixed‐effect model and two‐way ANOVA analysis. A *p* < 0.05 was considered statistically significant (Figure 1).

## 3 RESULTS

### 3.1 Amylase and lipase in metabolically normal monkeys: Effect of age

Metabolically normal monkeys were grouped into 4‐year age intervals, ranging from 4 to 23 years, to determine the effect of age on amylase levels, without the influence of metabolic dysfunction (Table 1). Amylase levels declined significantly with older age (after age 16–19 (*p* < 0.05) and oldest age group (20–23 years)) compared to all other metabolically normal age groups (*p* < 0.01) by linear mixed‐effect analysis. Serum lipase levels showed no significant differences between the age groups.

**TABLE 1:** Serum amylase and lipase levels in different age groups of metabolically normal rhesus monkey.

Age (years)	*N* monkeys	Amylase (U/L) (min–max)	Lipase (U/L) (min–max)
3–7	26	268.1 ± 62.1 (170–415)	31.6 ± 21.9 (10–107)
8–11	57	258.2 ± 59.1 (149–397)	32.3 ± 19.8 (8–93)
12–15	43	237.3 ± 55.1 (153–429)	49.6 ± 27.6 (11–135)
16–19	25	217.7 ± 68.9* (127–410)	35.2 ± 21.7 (10–106)
20–23	9	175.5 ± 39.8** (112–219)	48.5 ± 19.3 (21–84)

*Note*: Data are mean ± SD and minimum and maximum values. These age groups include only metabolically normal NHPs (excludes male monkeys with body weight >13.5 kg and females with body weight >8.5 kg). Further all male and females monkeys with body fat >22%, FPI >70μU/mL, FPG >80 mg/dL, HbA1c >4.5 and TG >150 mg/dL were excluded in these age groups. Note that monkeys entered the colony at different ages and only data while they remained metabolically normal are included here regardless of age. Some animals that remained normal are included in more than one age group.

* *p* < 0.05 Compared to age group 3–7.

** *p* < 0.01 compared to age group 3–7 and 8–11 by mixed model linear regression, multiple comparison by Bonferroni post hoc test.

### 3.2 Associations between amylase, lipase and metabolic parameters

Multivariable linear regression analysis revealed that amylase levels negatively predicted % body fat in model 1 (*β* −0.41; *p* < 0.001) with % body fat contributing about 16% to the variability of the amylase level. Age of the NHPs also negatively predicted 1 (*β* −0.28; *p* < 0.001) amylase levels in model 2. Adding age in the model 2 improved the prediction from 16 to 23% (*R* ^2^ change *p* = 0.003). Amylase levels were significantly predicted in model 3 by a combination of age and lipase levels accounting 27% variability by addition of 4% from model 2 (*p* = 0.023). Amylase showed a positive correlation with increase in lipase levels. Amylase levels were further predicted significantly by the combination of % body fat, age, lipase and HbA1c accounting 31% variability (*R* ^2^ change *p* = 0.037). Adding body weight, fasting blood glucose, fasting insulin, HDL cholesterol, and triglycerides does not showed variability in the amylase levels (Table 2). Lipase levels across the metabolic phases not shown any significant associations with metabolic parameters (data not shown).

**TABLE 2:** Multivariable linear regression analysis to predict amylase level (*n* = 157).

Predictors	Model 1 B (95%CI) β		Model 2 B (95%CI) β		Model 3 B (95%CI) β		Model 4 B (95%CI) β	
Body fat (%)	−2.6 (−3.7 to −1.5)	−0.41***	−1.9 (−3.1 to −0.7)	−0.30**	−2.1 (−3.2 to −0.8)	−0.32**	−1.9 (−3.0 to −0.7)	−0.29**
Age (years)			−4.0 (−6.6 to −1.3)	−0.28**	−4.4 (−7.0 to −1.8)	−0.30**	−3.7 (−6.4 to −1.1)	−0.27**
Lipase U/L					0.45 (0.06 to 0.85)	0.20*	4.4 (0.59 to 8.3)	0.19*
HbA1c							−0.10 (−19.9 to −0.61)	−0.18*
*R*	0.16***		0.23***		0.27***		0.31***	
*R* ^2^ Change	0.16***		0.07**		0.04*		0.03*	

*Note*: Adjusted for body weight (kg), fasting blood glucose, fasting insulin, insulin sensitivity, HDL and triglycerides.

Abbreviations: 95% CI, 95% confidence interval of B; B, unstandardized regression coefficient; *R*, portion of the variance in amylase that can be explain by variables in each model; *R* ^2^ Change, change in the portion of variance in amylase due to new variable added in the model; β, standardized coefficient.

* *p* < 0.5.

** *p* < 0.01.

*** *p* < 0.001.

### 3.3 Longitudinal analysis of amylase and lipase during the phases in the progression from normal to overt T2DM

**FIGURE 2:**
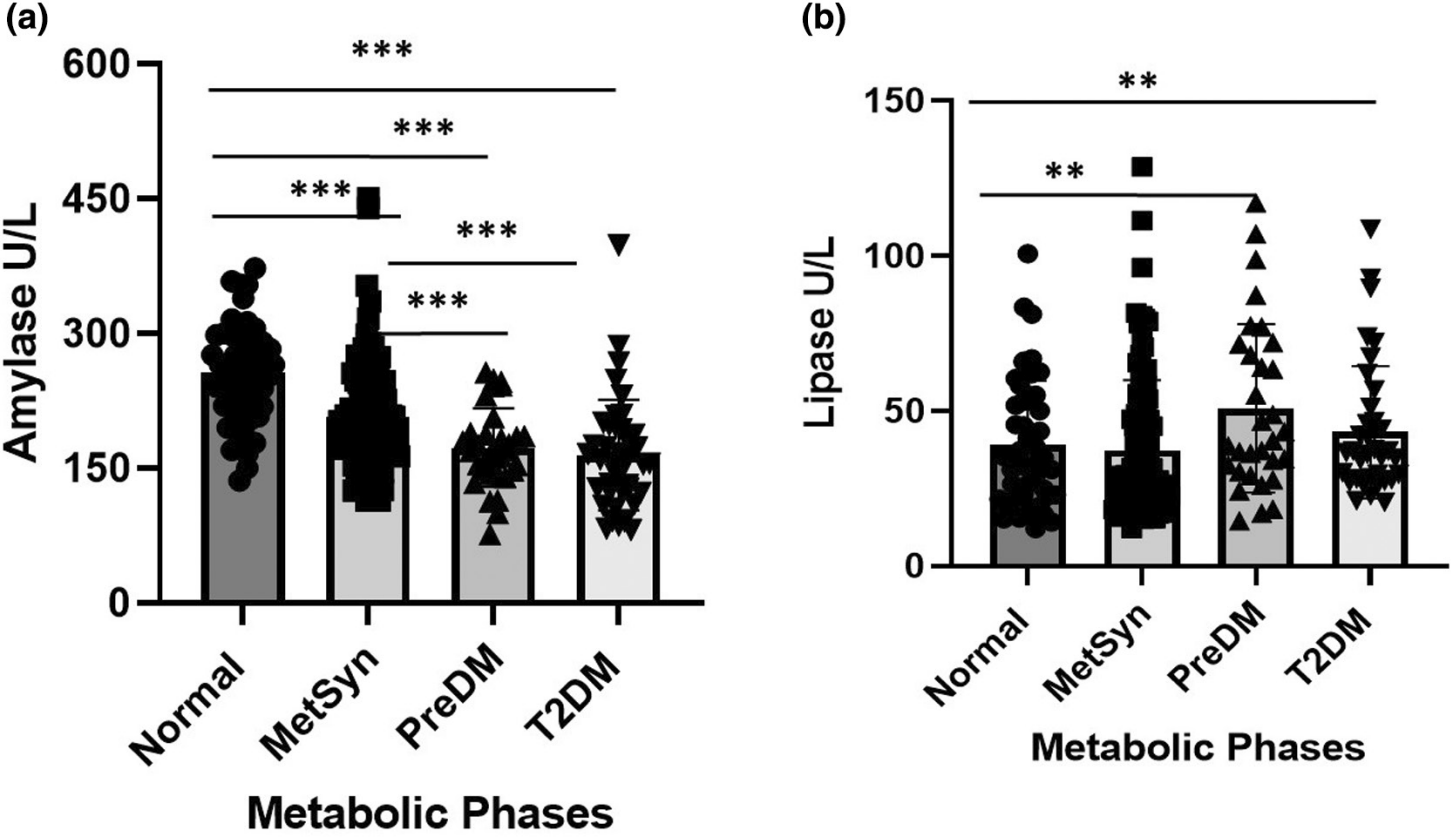
Cross sectional data of serum amylase and lipase in different metabolic groups: normal, MetSyn, PreDM, and T2DM. Amylase significantly (*p* < 0.001) decreased in MetSyn, PreDM and T2DM compared to normal monkeys (a). Lipase significantly increased in PreDM and DM compared to normal and MetSyn monkeys (b). **p < 0.01, ***p < 0.001 (Normal *n* = 51; MetSyn *n* = 44; PreDM *n* = 27; T2DM *n* = 35). Mixed model linear regression, multiple comparison by Bonferroni post hoc test.

The decline of amylase and increase of lipase over the progression from normal (phases 1–3) to metabolic syndrome (phases 4–6), PreDM (phase 7) and overt T2DM (phase 8) are shown in Figure 2. Grouping monkeys by their metabolic stage revealed differences in both the amylase and lipase as shown in Figure 2. Metabolically normal monkeys had significantly higher amylase than those with metabolic syndrome (*p* < 0.001), PreDM (*p* < 0.001), or T2DM (*p* < 0.001). Metabolic syndrome monkeys had higher amylase than those with PreDM and T2DM (both *p* < 0.001) (Figure 2a). Metabolically normal monkeys had lower lipase than monkeys with PreDM (*p* = 0.002) and T2DM (*p* = 0.004). (Figure 2b).

### 3.4 Amylase and triglyceride levels are related to age at T2DM onset

**FIGURE 3:**
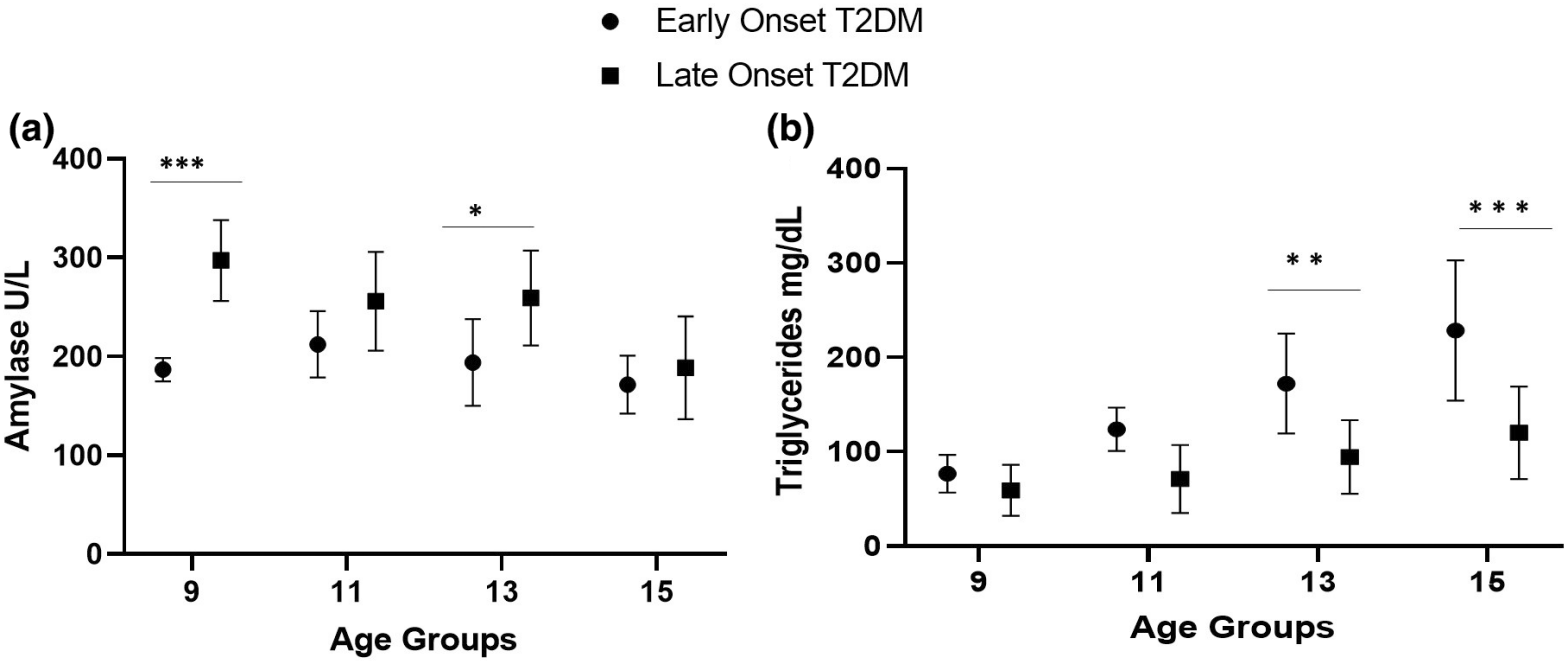
Longitudinal data showing amylase and TG trajectories in early onset (*n* = 7) and late onset T2DM (*n* = 8). Amylase showing decline trajectory and triglyceride showed increase trajectory (b) before onset of T2DM in the early age onset T2DM compared to late age onset T2DM. The average early age of onset was 14.2 ± 2.0 years and 18.5 ± 3.2 years in late age onset T2DM in NHPs. *p < 0.05, **p < 0.01, ***p < 0.001 by mixed model two‐way ANOVA. (a) amylase has triple stars which are all *p* < 0.001 (b) both are ** *p* < 0.01.

Monkeys that developed T2DM while under study were evaluated to identify the relationship between amylase and TG and the timing of T2DM onset. Amylase levels were lower at age 9 years (*p* < 0.001) and 13 (*p* = 0.03) in early onset (average age 14.2 ± 2.0 years) T2DM compared to late onset (18.5 ± 3.2 years) T2DM group. However, TG was significantly higher in the early onset of T2DM at 13 (*p* < 0.006) and years (*p* < 0.001) 15 years compared to the late onset T2DM (Figure 3a,b).

## 4 DISCUSSION

### 4.1 Normal serum amylase and lipase levels in rhesus monkeys

The range of amylase observed in the metabolically normal monkeys was 79–540 U/L. The upper limit of amylase was substantially higher than most reports of human patients. Studies in human adults have reported amylase ranges in various populations such as in Korean (56–107 U/L) (Lee et al., 2011), Japanese (31–193 U/L) (Nakajima et al., 2011), Chinese (11–142 U/L) (Zhuang et al., 2016), and the USA (48–86 U/L) (Dias et al., 2016). Due to changes in the amylase and lipase assays around 1990, reports preceding this date are not comparable to results reported after 1990. Serum amylase for all monkeys in the present study, including those with diabetes, was 240.84 ± 2.6 (range 66–658 U/L) and was in agreement with the prior reports of limited number of young adult male rhesus monkey amylase levels by Chen et al (Chen et al., 2010). (385 ± 145 U/L) and Blakely et al (Blakely et al., 2007). (267 ± 16.6 U/L). Baboon (Papio) and chimpanzees (Pan) do not have cheek pouches, leading to speculation that this anatomy might be related to the lower levels of amylase in these species (Lambert, 1998). Salivary amylase gene Copy Number Variations (CNVs) and amylase protein expression have been observed in high starch eating populations compared to low starch eating human populations (Perry et al., 2007).

An age‐related decline in serum amylase in monkeys (≥20 years) was observed in this study. This study reporting the amylase and lipase levels across a large cohort of NHPs across different age groups and their metabolic status. Salivary amylase has also been reported to decrease with age in rats (Kim, 1981; Mahay et al., 2004). In humans, Ben et al (Ben–Aryeh et al., 1986). found significantly lower amylase activity in the saliva of older individuals. Studies in aged rats (Khalil et al., 1985) and in humans over 65 years reported a significant reduction in pancreatic amylase secretion and pancreatic amylase enzyme concentrations (Laugier et al., 1991; Vellas et al., 1988). Decreased levels of both salivary and pancreatic amylase secretion may have contributed to the lower levels of serum amylase in the advanced‐aged cohort (≥20 years) of monkeys reported in this study. We did not; however, assess the distribution of the different sources of amylase for, example, salivary versus pancreatic levels. A 1986 report stated that serum amylase is equally derived (1:1) from salivary and pancreatic sources (Mifflin et al., 1986).

Serum lipase levels have been reported in only one study involving five male rhesus monkeys aged 5–6 years with serum lipase levels of 25.8 ± 13 U/L (Chen et al., 2010). In the present cohort of metabolically normal monkeys of similar age the average lipase was 34.6 ± 4.9 U/L (mean ± SE; range 9–128 U/L), similar to the prior report.

Analysis across all monkeys with and without T2DM showed inverse relationships between amylase and increasing age and HbA1c. This study also established a pattern of decline in serum amylase in metabolically normal monkeys with advancing age and this decline was greater with the development of metabolic syndrome and T2DM.

A significant increase in serum lipase throughout the metabolic trajectory toward T2DM was observed in this cohort of NHPs. Previous studies in obese middle‐aged rats have shown increased lipase in plasma and pancreatic tissues (Bruzzone et al., 1984; Schneeman et al., 1983). Reports in humans have varied widely, with a 31% decrease in serum lipase in T1DM and a 30% decrease in T2DM patients (Aughsteen et al., 2005; Madole et al., 2016), and an increase of 13% (Malloy et al., 2012) and 16.6% (Steinberg et al., 2014) in patients with T2DM.

### 4.2 Effect of obesity and insulin resistance on serum amylase and lipase

Linear regression analysis showed a negative correlation of increased adiposity with decreased amylase levels. An earlier study in obese rats found that pancreatic amylase activity decreased in obese and remain elevated in lean rats (Schneeman et al., 1983). Few studied in children showed lower amylase levels in obese associated with low copy number of salivary AMY1 gene (Locia‐Morales et al., 2022; Vázquez‐Moreno et al., 2020). No other clinical studies have investigated the relationship between amylase and obesity. In the present study % body fat, as an indicator of adiposity, negatively predicted amylase levels. The amylase levels declined longitudinally with progression of metabolic syndrome to T2DM.

Insulin is known to regulate amylase mRNA and exocrine pancreatic amylase production (Korc et al., 1981) and secretion (Patel et al., 2004). Young, obese hyperinsulinemic rats showed an increase in pancreatic amylase production (Trimble et al., 1986), although, in aged, obese rats, hyperinsulinemia resulted in a lower level of pancreatic amylase, and impairment of amylase gene expression (Trimble et al., 1986). In the present study, normal insulin levels during the normal metabolic phases 1 and 2, were associated with normal amylase levels; however, the progression of metabolic syndrome (phases 4 to 6) showed an increase in insulin, but a decline in amylase. This observation is in agreement with Trimble et al., (Trimble et al., 1986) where aged rats with hyperinsulinemia showed lower amylase levels. Studies in a pig model described pancreatic amylase regulation for glucose homeostasis by enhancing intestinal glucose metabolism and lowering net glucose absorption and insulin secretion (Pierzynowska et al., 2018; Pierzynowski et al., 2017). In addition, lower serum amylase has been associated with decreased insulin and the development of insulin resistance in middle‐aged humans (Muneyuki et al., 2012). Also, consistent with the present study, showing a decline in amylase with the progression of metabolic syndrome in middle‐aged monkeys, Zhuang et al (Zhuang et al., 2016). reported that low amylase was associated with impaired islet β‐cell function in patients with early‐onset T2DM. Community‐based studies have also shown a decrease in amylase with an increase in plasma insulin and insulin resistance (Muneyuki et al., 2012), prior to the progression to overt diabetes. The present study showed decreased insulin sensitivity during the metabolic progression toward diabetes and was associated with a decrease in amylase in pre‐diabetic phase.

Lipase was observed to rise with increasing insulin levels and decreasing insulin sensitivity in PreDM and T2DM monkeys. A marginal increase in serum lipase was reported in 13% and 16% of patients with T2DM, respectively (Malloy et al., 2012; Steinberg et al., 2014). Three or more times the upper limit of normal serum lipase is the preferred diagnostic indicator of acute pancreatitis (Ismail & Bhayana, 2017; Yadav et al., 2002); however, the etiology and clinical significance of marginal increases during preDM and T2DM is unknown and warrants further investigation (there was no indication of acute pancreatitis in this NHP colony).

### 4.3 Amylase and triglyceride levels in T2DM

NHPs develop metabolic syndrome and T2DM in middle age that appears to be pathophysiologically similar to humans. Longitudinal evaluation of amylase, lipase, body weight and TG in a group of diabetic monkeys divided into either middle‐age onset or and later‐age onset showed a similar pattern of decrease in amylase levels throughout middle‐age. However, early onset T2DM monkeys showed significant increases in TG before T2DM and it appeared that decreased amylase and increased TG predisposed monkeys to the middle‐aged onset of diabetes compared to the later age onset of diabetes. Although monkeys with later age DM‐onset have had decreased amylase levels, they developed diabetes when the levels of TG were increased to the threshold similar to early onset monkeys. Prospective studies in human patients have also shown that increases in TG contribute to the risk of T2DM development (He et al., 2012; Tirosh et al., 2008). Low serum amylase and elevated TG, in combination, appear to be viable markers to improve the prediction of the development of T2DM in NHPs, and the same associations are likely to be predictive in human populations.

### 4.4 Limitations of the study

Because of the long, multi‐year trajectories of the monkeys in the current study (and of humans studied longitudinally), approximately 30% progressed through two or more of the established metabolic groups, further emphasizing the importance of high quality longitudinal studies. Further, amylase and lipase were measured following an overnight fast. Changes after feeding, or during intravenous glucose tolerance tests, or with euglycemic hyperinsulinemic clamps were not measured. Serum amylase and lipase levels during feeding, or in response to insulin, may be indicators of pancreatic exocrine secretion (Keller & Layer, 2005).

In conclusion, amylase was found to decrease, despite no change in lipase, with advancing age in metabolically normal monkeys. Amylase decreased even more significantly in monkeys with metabolic syndrome, PreDM, and T2DM. In contrast, lipase increased in PreDM and T2DM. A gradual decrease in insulin sensitivity during the progression of metabolic syndrome/PreDM and T2DM in monkeys was associated with the decreases in amylase and increases in lipase. Low amylase levels before the onset of T2DM and high triglycerides predisposed monkeys to early onset of T2DM. This is the first extensive report showing the longitudinal trajectories of amylase and lipase over a wide range of ages and metabolically diverse monkeys. Such trajectories are not available for any other species, including humans.

## AUTHOR CONTRIBUTIONS

UKC was involved in the literature search, data analysis, writing, and editing of the manuscript; BCH developed and oversaw the study and all laboratory personnel and procedures, contributed to the content and analysis, contributed to the writing, literature review, and editing and critically revised the manuscript.
